# Utilizing Nanobody Technology to Target Non-Immunodominant Domains of VAR2CSA

**DOI:** 10.1371/journal.pone.0084981

**Published:** 2014-01-21

**Authors:** Sisse B. Ditlev, Raluca Florea, Morten A. Nielsen, Thor G. Theander, Stefan Magez, Philippe Boeuf, Ali Salanti

**Affiliations:** 1 Centre for Medical Parasitology at Department of International Health, Immunology, and Microbiology, University of Copenhagen and at Department of Infectious Diseases, Copenhagen University Hospital (Rigshospitalet), Copenhagen, Denmark; 2 Cellular and Molecular Immunology Research Unit, Vrije Universiteit Brussel, Brussels, Belgium; 3 Department of Structural Biology, VIB, Brussels, Belgium; 4 The University of Melbourne, Department of Medicine, Parkville, Victoria, Australia; 5 Victorian Infectious Diseases Service, Royal Melbourne Hospital, Parkville, Victoria, Australia; Institut national de la santé et de la recherche médicale - Institut Cochin, France

## Abstract

Placental malaria is a major health problem for both pregnant women and their fetuses in malaria endemic regions. It is triggered by the accumulation of *Plasmodium falciparum*-infected erythrocytes (IE) in the intervillous spaces of the placenta and is associated with foetal growth restriction and maternal anemia. IE accumulation is supported by the binding of the parasite-expressed protein VAR2CSA to placental chondroitin sulfate A (CSA). Defining specific CSA-binding epitopes of VAR2CSA, against which to target the immune response, is essential for the development of a vaccine aimed at blocking IE adhesion. However, the development of a VAR2CSA adhesion-blocking vaccine remains challenging due to (*i*) the large size of VAR2CSA and (*ii*) the extensive immune selection for polymorphisms and thereby non-neutralizing B-cell epitopes. Camelid heavy-chain-only antibodies (HcAbs) are known to target epitopes that are less immunogenic to classical IgG and, due to their small size and protruding antigen-binding loop, able to reach and recognize cryptic, conformational epitopes which are inaccessible to conventional antibodies. The variable heavy chain (VHH) domain is the antigen-binding site of camelid HcAbs, the so called Nanobody, which represents the smallest known (15 kDa) intact, native antigen-binding fragment. In this study, we have used the Nanobody technology, an approach new to malaria research, to generate small and functional antibody fragments recognizing unique epitopes broadly distributed on VAR2CSA.

## Introduction

Placental malaria is caused by the protozoan *Plasmodium falciparum* transmitted by the female Anopheles mosquito and can lead to maternal anemia, low birth weight, preterm delivery and increased infant and maternal mortality. *P. falciparum*-infected erythrocytes (IE) accumulate in the placenta by adhering to chondroitin sulfate A (CSA) chains on chondroitin sulfate proteoglycans (CSPG) in the intervillous spaces and on the microvillous membrane of the placental syncytiotrophoblast [1]. IE adhesion is mediated by VAR2CSA, a pregnancy-specific member of the *P. falciparum* erythrocyte membrane protein 1 (PfEMP1) family expressed on the surface of IE [2]. In malaria endemic areas, children develop clinical immunity through the acquisition of a broad repertoire of anti-PfEMP1 antibodies [3]. Pregnant women become susceptible to malaria, as they have not previously acquired antibodies to the pregnancy-specific PfEMP1 variant VAR2CSA. IE adhesion to the placenta triggers the recruitment and activation of maternal mononuclear cells secreting pro-inflammatory cytokines, leading to further inflammation and negative effects on placental function [4] and fetal development [5]. During subsequent pregnancies, women build up protective immunity to placental malaria by acquiring anti-VAR2CSA antibodies that prevent IE binding to CSA in the placenta [6], [7]. VAR2CSA is therefore an attractive candidate for a vaccine against placental malaria. VAR2CSA is a large protein (∼350 kDa) consisting of six Duffy-Binding-Like domains and several inter domains [8], [9]. Even though VAR2CSA is conserved relative to other PfEMP1 proteins, there is a substantial sequence variation [10]. Thus, a major challenge for vaccine development is to define VAR2CSA epitopes that can induce a broad anti-adhesive antibody response. Several single domains of VAR2CSA have been shown to be able to induce functional adhesion-blocking antibodies by immunization in laboratory animals, even though these domains do not directly take part in VAR2CSA binding to CSA [11]–[17]. Recent studies have highlighted the importance of the N-terminal part of VAR2CSA in CSA-binding and antibodies targeting this region effectively prevent VAR2CSA binding to CSA [18]–[20]. However, identification of smaller VAR2CSA regions responsible for CSA binding is a major challenge since VAR2CSA is a large and complex antigen. The identification of such epitopes could pave the way towards designing an effective multivalent VAR2CSA vaccine. We have extensively explored the naturally-acquired response to VAR2CSA in order to differentiate the protective adhesion-blocking response from the immuno-dominant, non-functional response focused towards the DBL3X, DBL5ε and DBL6ε domains of VAR2CSA [21], [22]. Indeed, the majority of the naturally-acquired response targets the C-terminal part of VAR2CSA that does not mediate binding to CSA [22]. The majority of hybridomas cloned from mice and rats immunized with full-length VAR2CSA produced IgG against DBL3X and DBL5ε domains and these antibodies did not block IE adhesion to CSA (unpublished data).

In this study, we introduce an approach new to malaria research to produce versatile and functional monoclonal reagents against VAR2CSA circumventing IgG immuno-dominant epitopes, based on camelid heavy-chain-only antibodies (HcAbs). The variable heavy chain (VHH) domain is the antigen-binding site of camelid HcAbs and represents the smallest (15 kDa), intact, native antigen-binding fragment [23]. Recombinantly-produced VHHs are termed Nanobodies (Nbs). Nbs are easily expressed in large quantities, are soluble, have high thermal stability, and bind the target antigen with the *high affinity and specificity typical of conventional antibodies* [24]. Due to their small size and protruding antigen-binding complementarity determining region-3 (CDR3) loop, Nbs have the capacity to reach and recognize cryptic, conformational epitopes that are inaccessible to conventional antibodies [25]–[27]. Moreover, Nbs generally interact with epitopes that are less antigenic as compared to conventional antibodies [28]. These properties make them potent alternatives to conventional antibodies for non-immuno-dominant epitopes.

To investigate the potential of Nbs as a tool for targeting VAR2CSA, an alpaca was immunized with full-length VAR2CSA and the Nbs generated were screened for VAR2CSA-specificity and functionality. Using this approach, we produced a large panel of VAR2CSA-specific Nbs targeting epitopes broadly distributed over VAR2CSA, including against the poorly immunogenic CSA-binding regions. This study highlights the advantages of using the Nanobody technology for production of monoclonal reagents to pathogenic antigens that have evolved immuno-dominant regions.

## Results

### Library screening and selection of VAR2CSA-specific Nanobodies (Nbs)

**Figure 1:**
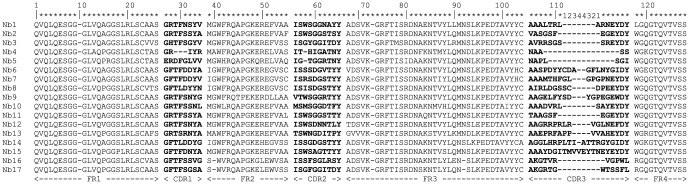
Sequence alignment of the 17 VAR2CSA-specific Nbs. The clones used for Nanobody production were sequenced, converted to amino acid sequences and aligned. The complementarity determining regions (CDRs) 1–3 make up the binding paratope and the framework regions (FRs) 1–4 are indicated.

A Nanobody phagemid library with a size of 1.7×10^8^ colonies was generated by cloning HcAbs from peripheral blood mononuclear cells of the VAR2CSA immunized alpaca. An aliquot of the library was infected with M13K07 helper phages and phages expressing VAR2CSA-specific VHHs were enriched by three consecutive rounds of bio-panning on VAR2CSA. From the second and third rounds of panning, 100 colonies randomly chosen were screened for VAR2CSA recognition by periplasmic extraction, followed by ELISA. The majority (70%) of these clones were found to specifically bind VAR2CSA but not the control protein BSA. Nucleotide sequence analysis of these clones revealed 17 genetically distinct VAR2CSA binders. Their paratope (CDR-3 region) amino acid sequences differed by several amino acids (Figure 1).

### Expression and purification of anti-VAR2CSA Nbs

The VHH vectors encoding the 17 VAR2CSA-specific Nbs were sub-cloned into the pHEN6c expression vector containing a C-terminal His_6_ tag. Nbs were expressed in WK6 *E. coli* cells and purified using HisTrap columns. The production yields of each Nb varied from 4 mg to 11 mg per litre culture. The SDS PAGE analysis of the purified Nbs showed no impurities after the purification steps and only showed formation of dimers in the Nb03 production. (Figure S1).

### Nanobody reactivity to recombinant VAR2CSA protein

**Figure 2:**
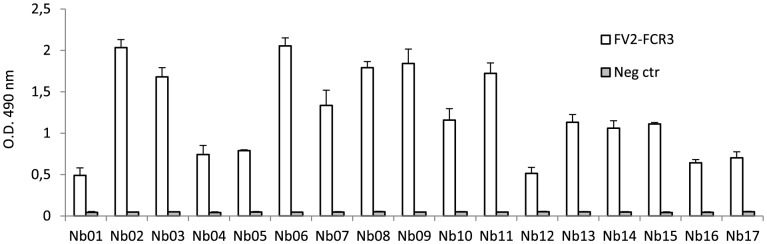
Recognition of immobilized full-length VAR2CSA by each of the 17 Nbs. Microtiter plates were coated with VAR2CSA protein (50 nM) and incubated with individual Nbs (50 nM). Binding was detected with rabbit anti-camel antibody and HRP-conjugated goat anti-rabbit antibody. Optical density was measured at 490 nm after 20 min. A non-VAR2CSA-PfEMP1 (50 nM) was used as negative control. Data are represented as mean and standard deviations of three independent experiments.

**Figure 3:**
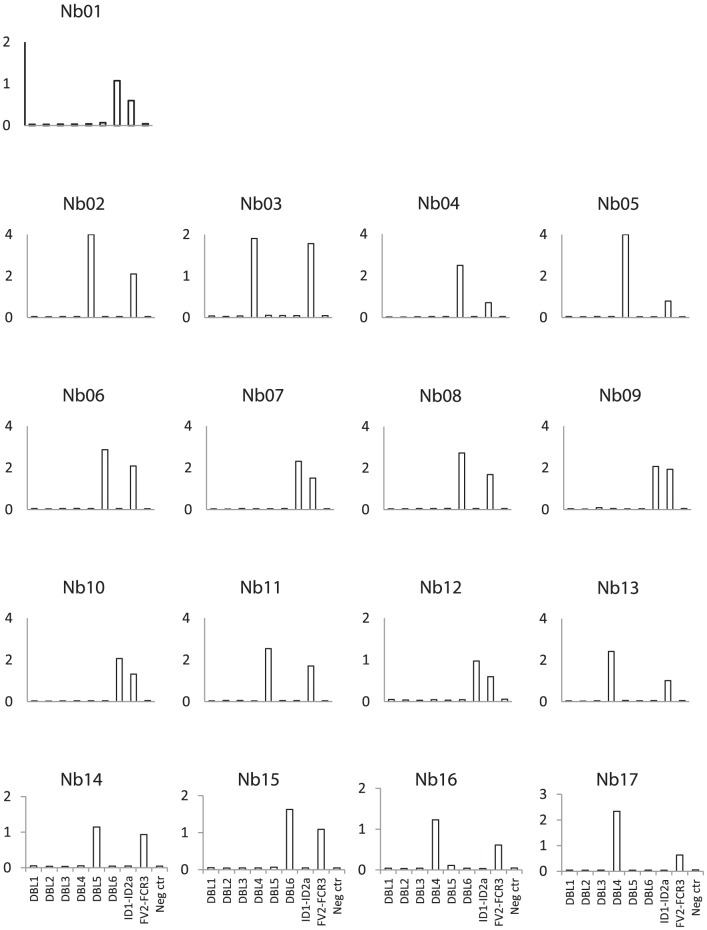
Recognition of immobilized VAR2CSA domains by Nbs (Nb01–Nb17). Baculovirus-produced domains of VAR2CSA (50 nM) were coated on microtiter plates and incubated with each of the Nbs (50 nM). The binding was detected with rabbit anti-camel and goat anti-rabbit HRP-conjugated antibodies and optical density measured at 490 nm after 20 min. Non-VAR2CSA PfEMP1 (50 nM) was used as negative control. The assay was performed several times with similar result.

To verify the specificity of the purified Nbs (Nb01–Nb17), direct binding to different domains of recombinant VAR2CSA was analyzed by ELISA (Figure 2). All 17 Nbs specifically recognized full-length VAR2CSA (FV2) and not a non-pregnancy specific PfEMP1 used as negative control. The Nbs were subsequently screened against individual VAR2CSA domains (DBL1-6) and against the ID1–ID2a region, which represents the minimal-CSA binding region of VAR2CSA [18], [20] (Figure 3). These domains were produced using a baculo-virus expression system, as described in [11], [20]. Table 1 shows an overview of the VAR2CSA domain recognition by the individual Nbs. The three C-terminal domains (DBL4-6) were recognized by twelve of the Nbs (71%). None of the Nbs recognized the individual N-terminal domains (DBL1-3) of VAR2CSA whereas five Nbs (Nb01, Nb07, Nb09, Nb10 and Nb12) recognized the ID1–ID2a minimal CSA-binding domain expressed in the baculovirus expression system.

**Table 1:** Summary of the reactivity of the individual Nbs against VAR2CSA (full length and domains).

	FV2-FCR3	ID1–ID2a	DBL1	DBL2	DBL3	DBL4	DBL5	DBL6
Nb01	X	X						
Nb02	X						X	
Nb03	X					X		
Nb04	X							X
Nb05	X						X	
Nb06	X							X
Nb07	X	X						
Nb08	X							X
Nb09	X	X						
Nb10	X	X						
Nb11	X						X	
Nb12	X	X						
Nb13	X					X		
Nb14	X						X	
Nb15	X							X
Nb16	X					X		
Nb17	X					X		

A cross in the box indicates specific binding.

**Figure 4:**
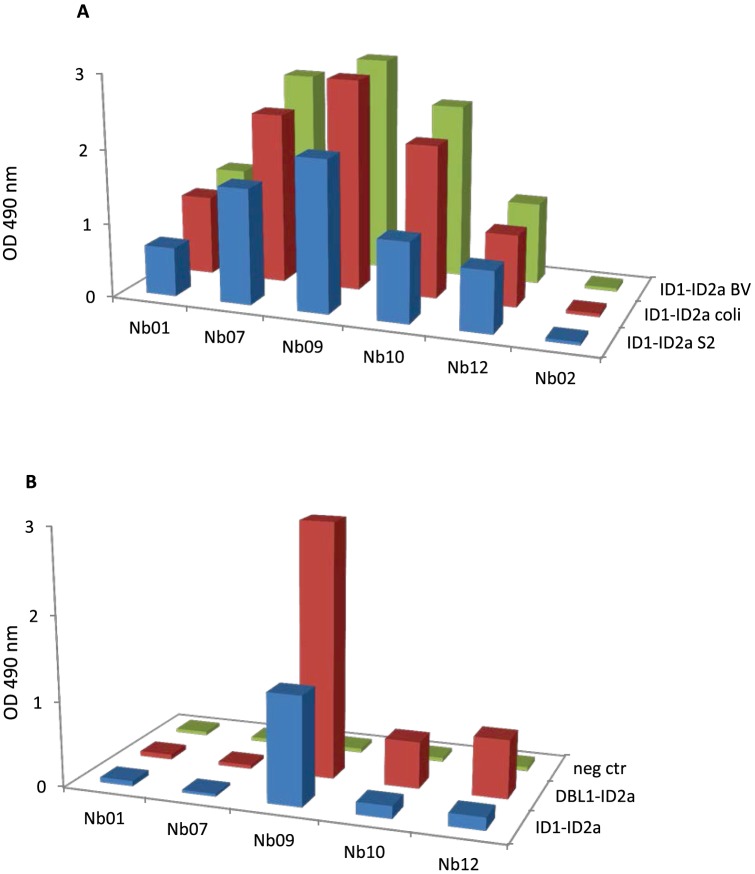
Recognition of the minimal CSA-binding region of VAR2CSA. (A) Reactivity of Nbs to specific minimal CSA-binding regions produced in different expression systems. ID1–ID2a proteins produced either in a baculo–virus expression system (BV), or in *E. coli* (coli) or in Schneider 2 (S2) cells were coated (50 nM) on microtiter plates and incubated with 50 nM Nb01, Nb07, Nb09, Nb10 or Nb12 (Nbs specific for VAR2CSA minimal CSA-binding domain). Nb02 (DBL5-specific) was used as a negative control. (B) Cross-reacitivity of Nbs specific for the minimal CSA-binding region of FCR3 to recombinant proteins covering the minimal CSA-binding region (DBL1-ID2a and ID1–ID2a) of the heterologous 3D7 parasite line produced in the baculovirus expression system. In both assays, the binding was detected with rabbit-anti-camel antibody and HRP-labeled goat anti-rabbit antibody and the optical density was measured at 490 nm after 20 min. A non-VAR2CSA protein was used as a negative control.

These five Nbs recognized to a similar degree ID1–ID2a minimal CSA-binding domains expressed in *E. coli* and S2 cells (Figure 4A). When tested against the minimal CSA-binding region of a heterologous parasite strain (3D7), three of these five Nbs (Nb09 and to some degree Nb10 and Nb12) were found to be cross-reactive (Figure 4B).

**Figure 5:**
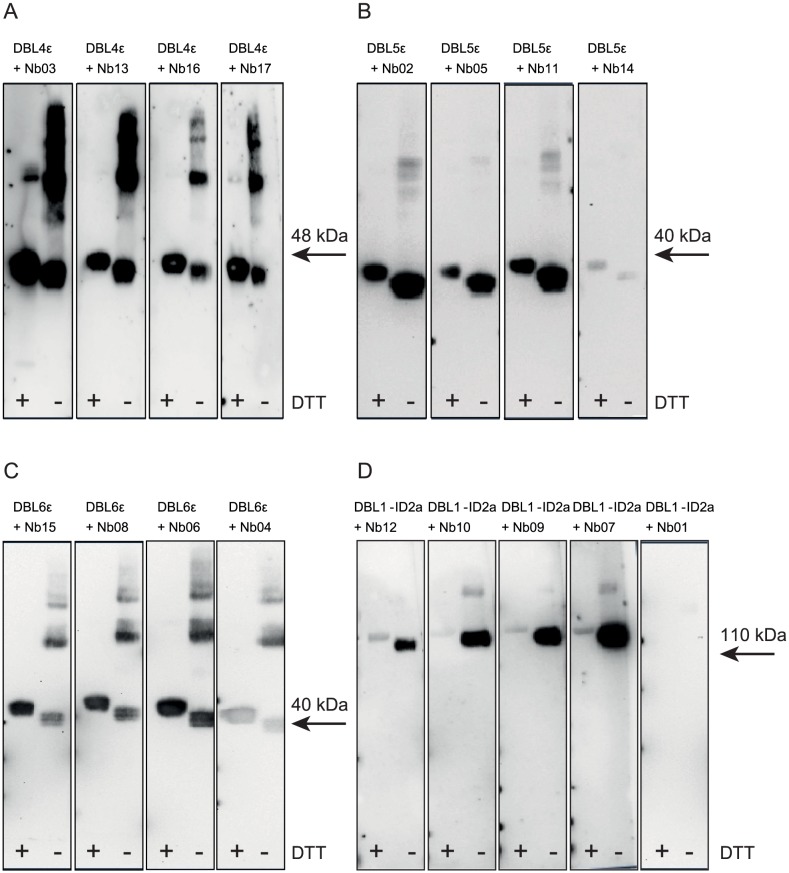
Nbs recognize both continuous and discontinuous epitopes. Reduced and non-reduced baculovirus-produced VAR2CSA domains were transferred onto membranes by Western blotting and probed with the corresponding domain-specific Nbs; (A) DBL4ε protein probed with Nb03, Nb13, Nb16 and Nb17. (B) DBL5ε protein probed with Nb02, Nb05, Nb11, Nb14. (C) DBL6ε probed with Nb15, Nb08, Nb06 and Nb04. (D) DBL1-ID2a protein (containing minimal CSA-binding region) probed with Nb12, Nb10, Nb09, Nb07 and Nb01. Binding was detected with rabbit-anti-camel and HRP-labeled goat-anti-rabbit antibodies. Bands larger than monomer size correspond to multimers formed by intermolecular disulfide bonds (monomers are marked with arrows).

We tested whether the epitopes recognized by the 17 Nbs were discontinuous using Western Blotting of reduced or non-reduced recombinant VAR2CSA protein. The Nbs specific for single domains showed similar binding to both the reduced and the non-reduced recombinant protein (Figure 5A–C), whereas the minimal CSA-binding region-specific Nbs showed no or very limited reactivity to the reduced protein (Figure 5D).

### Nanobody reactivity to native VAR2CSA protein expressed on the surface of IE

**Figure 6:**
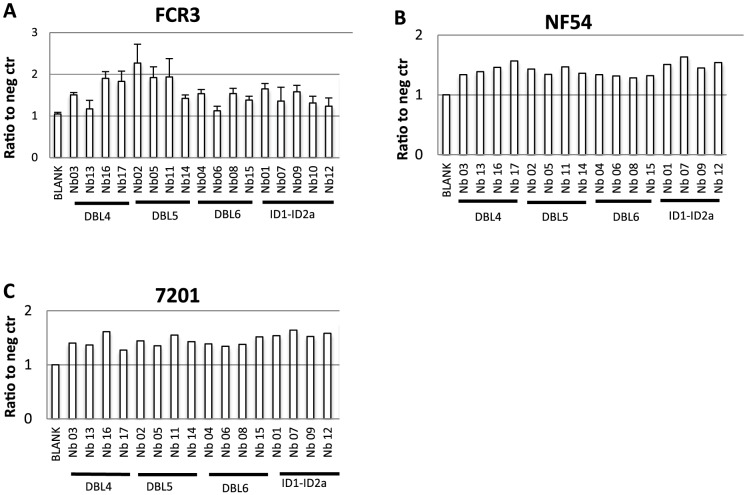
Nanobody recognition of native VAR2CSA expressed on the surface of *P. falciparum*-infected erythrocytes (IE). Binding of VAR2CSA-specific Nbs to VAR2CSA-expressing IE was measured by flow cytometry. Three different *P. falciparum* strains (FCR3, 7201 and NF54) were tested. Each bar represents reactivity of 25 µl nanobody (0.1 mg/ml) to 50 µl IE (2×10^5^ parasites/ml). Values are normalized to the mean fluorescence intensity (MFI) of the negative control (neg ctr; IE incubated only with the secondary and tertiary antibodies). Specific recognition was defined by an MFI ratio >1.2. Error bars in A) represents standard deviation of three independent experiments.

Epitopes exposed on recombinant proteins may not be surface-exposed on the native VAR2CSA protein expressed by IE. Therefore, we used flow cytometry to test the reactivity of the Nbs to VAR2CSA-expressing parasite lines (Figure 6). All Nbs showed (except Nb06 and Nb13) some degree of reactivity to VAR2CSA-expressing IE, including the homologous parasite line FCR3 and two heterologous parasite lines.

### Nanobody-mediated inhibition of IE binding to CSA

**Figure 7:**
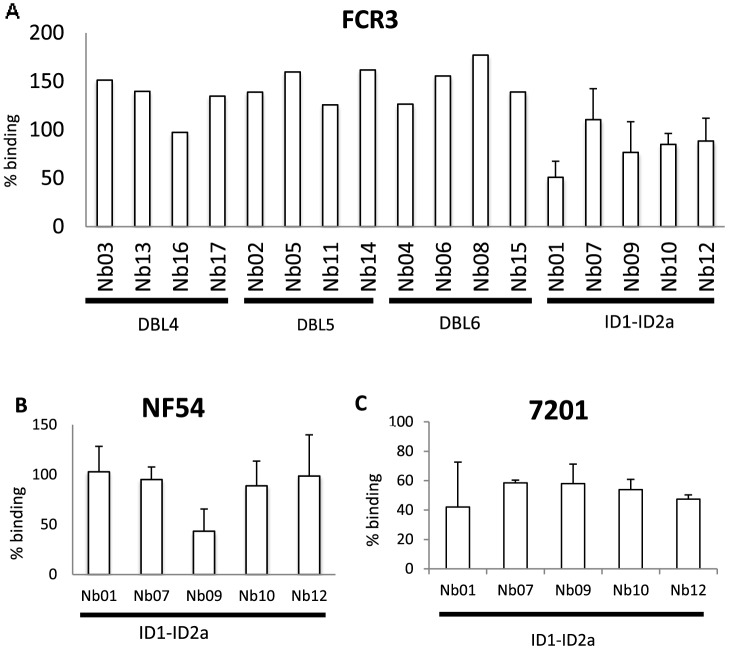
Adhesion-inhibitory capacity of VAR2CSA-specific nanobodies. (A) Ability of the individual Nbs to inhibit the adhesion of VAR2CSA-expressing IE (FCR3 line) to Decorin. Nbs specific for the minimal CSA-binding-region of VAR2CSA (Nb01, Nb07, Nb09, Nb10 and Nb12) were tested three times on the homologous parasite FCR3-CSA. (B) Adhesion inhibition capacity of the minimal CSA-binding-specific Nbs to VAR2CSA-expressing heterologous parasite lines NF54 and (C) 7201. Parasite binding to CSA ligand without Nbs was set to 100.

We evaluated the capacity of the Nbs to inhibit the adhesion of VAR2CSA-expressing IE to the placental receptor chondroitin sulfate A (Decorin) (Figure 7). Most Nbs increased IE adhesion to CSA but Nb01, Nb09 and Nb10, specific for VAR2CSA minimal CSA-binding region reproducibly inhibited CSA adhesion of the homologous FCR3 parasite line (15%–49% mean adhesion inhibition). The cross-inhibitory activity of the Nbs specific for VAR2CSA minimal CSA-binding region was assessed using two heterologous parasite lines (NF54 and 7201). All the Nbs specific for VAR2CSA minimal CSA-binding region reduced 7201 IE adhesion to CSA by at least 42% whereas NF54 IE adhesion to CSA was only inhibited by Nb09 (57% adhesion inhibition).

## Discussion

Identification of VAR2CSA epitopes that are target of protective antibodies is key to the development of multivalent vaccines that can protect pregnant women against placental malaria. However, the mapping of such epitopes has been hampered by the large and complex nature of VAR2CSA and the poor understanding of its interaction with the placental receptor CSA. Production and isolation of monoclonal antibodies to VAR2CSA from malaria-exposed women or VAR2CSA-immunized animals has been limited to the immuno-dominant DBL3X and DBL5ε domains [22], [29]. Because HcAbs can recognize poorly immunogenic epitopes [30] we hypothesized that HcAbs generated against VAR2CSA could circumvent the immuno-dominance of epitopes of the DBL3X and DBL5ε domains and induce a response against other VAR2CSA domains.

We immunized an alpaca with full-length VAR2CSA (FV2) and selected seventeen VHHs that specifically recognized FV2. This approach avoided a focused response towards the DBL3X and DBL5ε immuno-dominant domains since none of the Nbs targeted DBL3X and some Nbs recognized the less immunogenic CSA-binding N-terminal region of VAR2CSA. The twelve Nbs specific for the three C-terminal domains (DBL4ε, DBL5ε and DBL6ε) recognized these as single domains, whereas the five Nbs recognizing the N-terminal region did not react with single domains but was dependent on the entire minimal-CSA binding region, ID1–ID2a, encompassing DBL2X with the flanking parts ID1 and ID2. These results suggest that Nbs specific for the minimal CSA-binding region target discontinuous epitopes. This is in line with Western blot data showing that C-terminal-specific Nbs recognized linear epitopes whereas the N-terminal-specific Nbs recognize discontinuous epitopes. Taken together, these results support the proposed overall fold of VAR2CSA, in which the C-terminal domains are presented as single and accessible domains whereas the N-terminal domains containing the minimal CSA-binding region have a more complex conformation.

Nb01, Nb09, Nb10, and Nb12, which specifically recognize the minimal-CSA binding region of VAR2CSA, were found to partially block binding of VAR2CSA to CSA Importantly, Nb09 was both cross-inhibitory and cross-reactive since it both recognized the NF54 parasite and the recombinant VAR2CSA from the 3D7 strain (which has identical VAR2CSA sequence as the NF54 parasite) and reduced the NF54 parasite binding. The level of recognition was generally lower for the minimal CSA-binding region-specific Nbs than for Nbs recognizing the C-terminal domains. This could indicate that the epitopes recognized by these Nbs specific for the minimal CSA-binding region are more hidden in the structure, making it physically difficult for the secondary antibodies to bind the Nbs.

The CDR regions of Nbs are often longer and more flexible than corresponding regions of conventional antibodies and thus can bind epitopes that physically cannot be targeted by conventional immunoglobulins [28], [31], [32]. This could explain the concordance between reactivity to recombinant protein and to native VAR2CSA. The fact that Nbs can “penetrate” and bind hidden epitopes could stabilize the minimal CSA-binding region and facilitate crystallization, which has proven to be very challenging.

Natural selection of pathogen-derived antigens is associated with epitopes of varying immunogenicity, and it is likely that epitopes of functional importance will have evolved to avoid host antibody response. We demonstrated that the nanobody technology, through its capacity to recognize non-immuno-dominant and hidden epitopes, produces versatile monoclonal reagents to such antigens. Besides being used for quality control of VAR2CSA vaccine construct, VAR2CSA-specific Nbs could be novel diagnostic or therapeutic tools and could provide novel insights into structure/function of this complex antigen. This is essential to the design of a multivalent VAR2CSA vaccine.

## Materials and Methods

### Immunization

An alpaca was immunized with purified full-length VAR2CSA (FV2) recombinant protein expressed as described in [33]. Immunization of the alpaca and a bleed of 50 ml were done by Alpa-Vet (www.alpa-vet.be) and the procedure was approved by the ethics committee of the Free University Brussels (file number 12-601-1). The peripheral blood lymphocytes were isolated from the 50 ml of blood of the immunized alpaca using Lymphoprep (Nycomed).

### Construction of the Nanobody library and selection of VAR2CSA-specific Nanobodies

The nanobody library was constructed as previously described by Conrath et al, 2001. Briefly, total mRNA was extracted from 2×10^7^ lymphocytes from which 50 µg mRNA was used for the synthesis of cDNA with oligodT primer. Using the cDNA as template, the fragments encoding for both the VH and VHH domains of camelid IgGs were amplified by PCR with the CALL001 and CALL002 primers [34]. The VHH gene fragment (600 bp) was separated from the VH genes (900 bp) by agarose gel extraction and re-amplified with the framework-1 and framework-4 primers [35] in order to introduce PstI and NotI restriction sites. The PCR fragments were cloned into the phage-display phagemid vector pHEN4 and electro-transformed in electro-competent *E. coli* TG1 cells. The VHH library was expressed on phage after infection with M13K07 helper phages (invitrogen). Phages expressing VAR2CSA-specific VHHs on their coat proteins were enriched via three consecutive rounds of panning on microtiter plates (Nunc MaxiSorp®) coated with FV2 (10 µg/well). From the second and third round of panning, individual colonies were selected, grown in Terrific Broth (TB) media and VHH expression was induced with 1 mM isopropyl β-d-thiogalactopyranoside (IPTG). Identification of *E.coli* TG1 clones expressing anti-VAR2CSA Nbs was performed by periplasmic extract ELISA.

### Expression and purification of selected nanobodies

Genes encoding VAR2CSA-specific VHH were sub-cloned into pHEN6c expression vector using BstEII and PstI restriction sites, between a *pelB* leader signal sequence (for VHH periplasmic transport) and a C-terminal hexa-histidine (His_6_) tag and transformed into WK6 *E. coli* cells. Production of the recombinant Nbs was done in shaker flasks by growing the cells in TB media supplemented with ampicillin (0.1 mg/ml), MgCl_2_ (2 mM) and glucose (0.1%). When optical density at 600 nm was between 0.6–0.8, Nbs expression was induced with 1 mM IPTG for 16 h at 28°C under agitation (200 rpm) overnight. Periplasmic extract proteins were released by osmotic shock. The recombinant Nbs were purified from the periplasmic extract using HisTrap HP columns (17-5247-01, GE Healthcare). The bound protein was eluted with 10 mM NaH_2_PO_4_ (pH 7.4), 500 mM NaCl, and 500 mM imidazole. Nbs purity and formation of disulfide bonds were verified by SDS-PAGE using NuPage Novex Bis-Tris mini gels (invitrogen).

### ELISA mapping of VAR2CSA-specific nanobodies

Nanobody recognition of recombinant VAR2CSA protein and domains was measured by ELISA. Recombinant VAR2CSA (50 nM in PBS) was coated on Nunc MaxiSorp® plates overnight at 4°C. Non-specific binding sites were blocked by incubating the plates with 5% skim milk in PBS for one hour at room temperature (RT). After three washes in PBS+0.05% Tween-20, Nbs diluted in 1% skim milk in PBS were added to the wells (50 nM) and incubated for 90 min at RT. The plates were washed three times in PBS+0.05% Tween-20 before polyclonal rabbit anti-camel Ab (kindly provided by B. Stijlemans [36]) was added for 1 h (non-commercial; diluted 1∶2000 in 1% skim milk in PBS.) After three washes in PBS+0.05% Tween-20, horseradish peroxidase-conjugated (HRP) anti-rabbit-IgG (DAKO, P0448) diluted 1∶2000 in 1% skim milk in PBS was added for 1 h. The plates were washed three times in PBS+0.05% Tween-20 and binding of Nb was visualized by adding o-phenylenediamine substrate. After 20 min, the HRP enzymatic reaction was stopped by adding 2.5 M H_2_SO_4_ and the optical density at 490 nm measured using an ELISA plate reader (VersaMax Molecular Devices).

### Western Blot Analysis

Western blot was performed to determine if the individual Nbs recognized continuous or discontinuous VAR2CSA epitopes. Recombinant VAR2CSA protein (0,4 µg/lane) was loaded on an 8–12% Bis-Tris SDS gel (invitrogen) under reduced or non-reduced conditions. The separated proteins were transferred to nitrocellulose membranes by wet blotting. After blocking non-specific sites with 5% skim milk in TBS+0.05% Tween 20 (TBST), the membranes were incubated for 1½ hour at RT sequentially with VAR2CSA-specific nanobody (diluted to 50 nM in TBST), rabbit polyclonal anti-camel Ab (diluted 1∶2000 in TBST) and HRP-conjugated goat anti-rabbit antibody (DAKO; P0448 diluted 1∶3000 in TBST). Between incubations, the membranes were washed three times with TBST and Nb recognition of VAR2CSA was detected by chemiluminescence.

### Nanobody reactivity to IE using flow cytometry

Nbs reactivity to VAR2CSA–expressing IE was measured by flow cytometry. Approximately 100,000 late stage IE (50 µl at 2×10^6^ IE/ml) labelled with ethidium bromide were incubated with 50 µl Nbs (0,1 mg/ml). Nanobody binding was detected by 50 µl mouse-anti-penta-His Ab (Qiagen; 34660 diluted 1∶100 in PBS) and a FITC-labelled anti-mouse Ab (Vector: FI-2000; diluted 1∶200 in PBS). Each labelling step was conducted for 30 min at 4°C. As a negative control, IE were incubated with the secondary and tertiary antibody only. Data from 5,000 IE were collected on a FC500 flow cytometer (Beckman Coulter). The mean FITC fluorescence intensity was determined using Winlist Software (Verity Software House).

### Adhesion-inhibition capacity of nanobodies

The adhesion-inhibition capacity of various Nbs was measured in a high throughput assay as previously described [37]. Briefly, 2×10^5^ tritium-labelled late stage IE, pre-incubated or not with Nbs, were added to a decorin-coated plate (Sigma; D8428: 2 µg/ml) for 90 min at 37°C. Unbound IE were washed away by re-suspension performed by a pipetting robot (Beckman Coulter) and the detection of adhering IE was determined by liquid scintillation counting on a Topcount NXT (Perkin-Elmer).

## Supporting Information

Figure S1 **Coomassie-stained SDS-PAGE of purified Nbs Nb01–Nb17.** A: example of expression and purification of two different Nbs (Nb04 in lanes 2–6 and Nb07 in lanes 8–12). Lanes 2 and 8: Total protein in periplasmic lysate as loaded onto a HIS-column non-reduced. Lanes 3 and 9: Run through after purification on a HIS-column non-reduced. Lanes 4 and 10: Column wash non-reduced, Lanes 5, 6, 11 and 12: HIS-purified nanobody with (Lanes 5 and 11) or without (Lanes 6 and 12) reducing agent DTT. Lanes 1 and 7 are molecular markers (Prosieve™Color Protein Marker, Lonza). Figure B and C show the 17 produced and purified nanobodies under non-reduced conditions.
(TIF)
