# Supplementary figures and images for: Utilizing Nanobody Technology to Target Non-Immunodominant Domains of VAR2CSA
Source: PLoS One. 2014 Jan 21;9(1):e84981. doi: 10.1371/journal.pone.0084981 (PMC3897377; doi:10.1371/journal.pone.0084981)

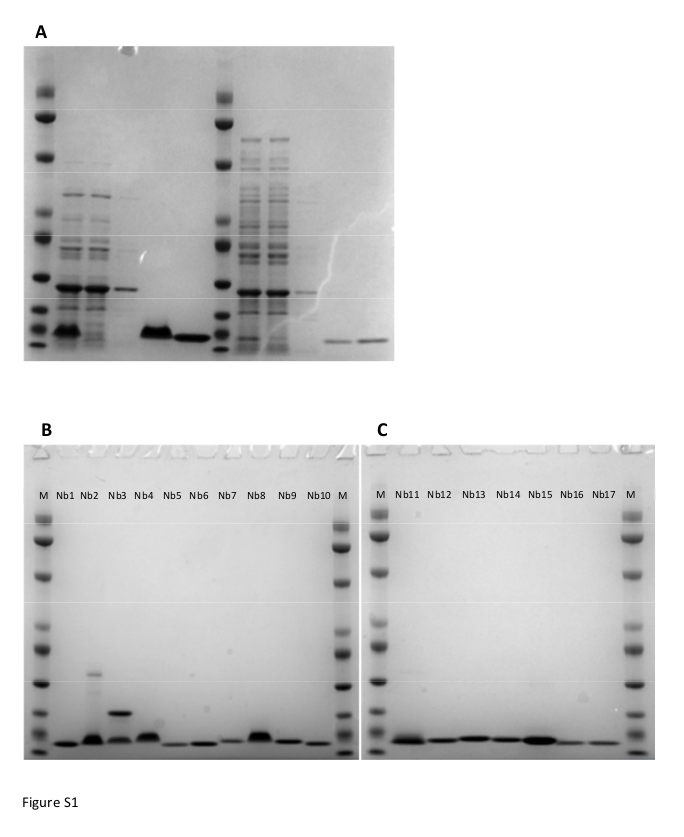

Supplement: Figure S1 — Coomassie-stained SDS-PAGE of purified Nbs Nb01–Nb17. A: example of expression and purification of two different Nbs (Nb04 in lanes 2–6 and Nb07 in lanes 8–12). Lanes 2 and 8: Total protein in periplasmic lysate as loaded onto a HIS-column non-reduced. Lanes 3 and 9: Run through after purification on a HIS-column non-reduced. Lanes 4 and 10: Column wash non-reduced, Lanes 5, 6, 11 and 12: HIS-purified nanobody with (Lanes 5 and 11) or without (Lanes 6 and 12) reducing agent DTT. Lanes 1 and 7 are molecular markers (Prosieve™Color Protein Marker, Lonza). Figure B and C show the 17 produced and purified nanobodies under non-reduced conditions. (TIF) [file pone.0084981.s001.tif]
